# Exploring the impact of self-efficacy on glycemic control in Omani type 2 diabetes patients

**DOI:** 10.3389/fendo.2025.1597274

**Published:** 2025-07-25

**Authors:** Ibrahim A. AlShezawi, Tamara H Al Rawwad, Ali A. Aldirawi, Abdallah A. Alwawi, Humoud S Al Fazari, Azmat H. Shah

**Affiliations:** ^1^ Nursing Department, Sultan Qaboos University Hospital, Suhar, Oman; ^2^ Department of Social Work, School of Applied Humanities and Social Sciences, German Jordanian University, Amman, Jordan; ^3^ Xiangya School of Nursing, Central South University, Changsha, Hunan, China; ^4^ Medical & Critical Care, Faculty of Health Professions, Al-Quds University, Abu Dies, Palestine; ^5^ Primary Health Care Department, Ministry of Health, Salalah, Oman; ^6^ Nursing Department, Pakistan Institute of Medical Sciences (PIMS), Islamabad, Pakistan

**Keywords:** self-efficacy, glycemic control, type 2 diabetes, Oman, diabetes self-management

## Abstract

**Introduction:**

Self-efficacy is critical for diabetic patients’ adherence to self-management behaviors, including medication compliance, diet modification, physical activity, and blood glucose monitoring.

**Objectives:**

This study assessed diabetes self-efficacy and glycemic control among adult patients with type 2 diabetes mellitus (T2DM) in Oman and identified determinants influencing glycemic regulation.

**Methods:**

A cross-sectional study was conducted from August to October 2023. Stratified cluster sampling was used to ensure regional representation. The governorate was divided into urban, suburban, and rural strata, from which 30 health institutions were randomly selected. This method allowed practical data collection and enhanced the reliability and generalizability of results. 225 adult T2DM patients were recruited from seven clinics and public hospitals in Oman. Data were collected through a self-administered questionnaire comprising socio-demographic, clinical characteristics, haemoglobin A1c levels, and the Self-Efficacy for Managing Chronic Disease 6 Scale, a validated tool measuring patients’ confidence in managing aspects of their chronic illness, including symptoms, treatment, and emotional distress. Inclusion criteria: adults aged 18 or older, clinically diagnosed with T2DM within the last year, and able to read Arabic. Exclusion criteria: individuals who declined participation, had severe physical illness at the time of the study, or psychological disorders. Statistical analyses were performed using SPSS version 29; multiple linear regression identified predictors of glycemic control.

**Results:**

Nearly half of the patients were aged 51–70 years, most were married, and over half were female. Approximately 50% had diabetes for over a decade. The mean hemoglobin A1c was 8.23 ± 2.08, indicating moderate glycemic control, while the mean self-efficacy score was 29.99 ± 11.41 out of 60 with cut-off point 30, suggesting low self-efficacy. Significant differences in glycemic control were observed by age (*p* = 0.015) and marital status (*p* = 0.025). Additionally, patients on both oral medications and insulin had poorer control (*p*< 0.001), whereas those with additional chronic diseases showed better control (*p* = 0.049).

**Conclusion:**

Low self-efficacy may contribute to inadequate glycemic control, while patients with comorbid conditions achieved better control, possibly due to heightened health vigilance and adherence. Research helps clarify these associations and informs interventions to enhance diabetes management.

## Introduction

Diabetes Mellitus (DM) is a chronic metabolic and endocrine disorder characterized by persistent hyperglycemia resulting from defects in insulin secretion and/or action (1). Type 2 Diabetes Mellitus (T2DM) is one of the most common chronic metabolic disorders worldwide. It is caused by impaired insulin signalling and increased hepatic glucose production. Over time, this dysfunction can lead to complications such as cardiovascular disease, nephropathy, neuropathy, and retinopathy (2). T2DM is associated with rapid urbanization and sedentary lifestyle changes (3). Globally, around 14% of adults are affected by diabetes mellitus. This figure is projected to rise to 46% by 2045 (1). More than 95% of these patients have type 2 diabetes (3, 4). The number is predicted to rise as a result of demographic shifts, rising obesity rates, lifestyle changes, and economic development (3, 4). Saudi Arabia exhibits the highest prevalence at 31.6%, followed by Oman at 29.0%, Kuwait at 25.4%, and Bahrain at 25.0% (5). In Oman, the increasing prevalence of diabetes is largely due to lifestyle changes. These include reduced physical activity, increased weight gain, and social factors that limit effective diabetes management (1, 6). In rural and mountainous areas, access to healthcare services and diabetes education programs is limited. This restriction hinders patients’ ability to manage their condition effectively (7).

Although T2DM is increasingly common in Oman, little is known about how well patients adhere to self-efficacy principles or maintain glycemic control. Previous research has predominantly focused on clinical outcomes or healthcare access for T2DM (8, 9); however, there is less understanding of the particular behavioral and socioeconomic factors that affect glycemic control among this population. Self-efficacy refers to an individual’s confidence in their ability to manage events and perform actions successfully. It is believed that self-efficacy plays a significant role in treatment compliance and therapeutic outcomes (10). According to Bandura’s Social Cognitive Theory, self-efficacy plays a central role in shaping decisions to adopt and sustain healthy behaviors (11) (9). Consequently, self-efficacy plays a crucial role in determining how well patients adhere to self-care behaviors, including medication adherence, diet modifications, physical activity, and keeping track of their blood glucose levels (12). Based on previous studies, self-efficacy enhances self-management practices, reduces diabetes complications, and improves glycemic control. Self-efficacy can be enhanced through structured diabetes education, goal-oriented interventions, and tailored behavioral coaching (8, 13).

Glycemic control pertains to the extent of elevated blood sugar levels experienced by an individual. Hyperglycemia can be diagnosed by doing blood glucose tests, such as fasting plasma glucose or HbA1c testing. Plasma glucose testing assesses the concentration of glucose in the blood, while HbA1c testing quantifies the quantity of glucose attached to red blood cells, giving an average blood glucose level over the past two to three months, although it is more reflective of the previous 30 days (14). In the majority of people with T2DM, glycemic control is usually regulated by a variety of self-management techniques such as dietary adjustments and physical exercise, weight reduction, and prescribed medications (15). Ensuring proper of glycemic control is crucial to prevent the onset of T2DM and minimize the associated consequences. The prevalence and management of glycemic control in T2DM differ across regions. These variations are influenced by economic conditions, population density, environmental factors, and dietary habits (16). Poor glycemic control may lead to symptoms of hyperglycemia. It can also cause complications such as delayed wound healing, dehydration, diabetic ketoacidosis, and diabetic coma (17). Another research study has indicated that only approximately 50% of diabetes patients achieve their desired HbA1c levels (18). An additional research study discovered a positive correlation between elevated levels of HbA1c and an elevated risk of stroke and various microvascular complications such as retinopathy, vitreous hemorrhage, and renal failure (19).

Several factors are associated with glycemic control, such as extended time duration with diabetes, and it was associated with suboptimal glycemic control. Additionally, lack of health insurance, elevated cholesterol levels, and the medications used for diabetes are associated with inadequate glycemic control (20). Engaging in optimal lifestyle behaviors, such as intense physical exercise and avoiding using substances, was found to be correlated with glycemic control management (21). Another literature has shown that weight loss can improve glycemic control by addressing defective insulin signaling, which is directly linked to excessive body fat accumulation. The impact of patients’ age on glycemic control is still a subject of T2DM, with additional factors such as financial stress, psychosocial problems, and education level (22).

Despite recent improvements in treatment, glycemic control remains suboptimal, with a significant proportion of patients experiencing deteriorating glycemic control and diminished self-efficacy. Nonetheless, research on self-efficacy and glycemic control in Oman remains scarce, leaving a critical gap in our perception of the behavioral and psychological factors that are vital to glycemic control. Although international research emphasizes the significance of health literacy, socioeconomic status, and demographic factors in glycemic control, comparable studies in Oman are scarce. This gap requires a thorough investigation of self-efficacy and glycemic control and its factors to guide culturally and geographically suitable treatments. The findings will contribute to the development of more focused intervention, systematic diabetes instruction, behavioral reinforcement, and personalized goals to strengthen self-efficacy and enhance glycemic control. Incorporating self-efficacy-based strategies into diabetes care models can improve glycemic outcomes, lower healthcare costs, and support more informed public health decisions. This study aimed to assess diabetes self-efficacy and glycemic control among adult patients with type 2 diabetes mellitus in Oman and to elucidate the primary determinants influencing glycemic regulation.

## Methods

### Study design and population

This descriptive, cross-sectional study was conducted among adult patients diagnosed with Type 2 Diabetes Mellitus in Oman from 10 August to 24 October 2023. We used a stratified cluster random sampling technique. First, the governorate was divided into three strata: urban, suburban, and rural. Then, healthcare facilities were randomly selected from each stratum. The healthcare institutions in North Al Batinah were chosen for this study owing to their established reputation within the community for delivering specialized and comprehensive diabetic treatment. The healthcare institutions in North Al Batinah Governorate, Oman, were chosen as the study’s site for various significant reasons. The region possesses a robust network of basic and secondary healthcare facilities that deliver specialized and comprehensive care for chronic illnesses, such as type 2 diabetes mellitus. These institutions are esteemed for their accessibility, quality of care, and robust involvement with the local community. Secondly, North Al Batinah encompasses a heterogeneous demographic and socio-economic population, providing an excellent context for analyzing disparities in diabetes self-efficacy and glycemic regulation. The incorporation of both urban and rural people facilitates a more representative sample and improves the generalizability of the findings to analogous areas in Oman and the wider Gulf region. The governorate’s active participation in chronic disease prevention programs and patient education campaigns offers a significant framework for evaluating real-world outcomes and pinpointing deficiencies in existing diabetes care techniques. This intentional choice amplifies the study’s significance and prospective influence on health policy and clinical practice in Oman. Initially, participants were recruited from two healthcare facilities: the Al Khaburah Extended Health Center and the Wadi Bani Umar Health Center. Subsequently, the roster of collaborating health centers was expanded to include the Wadi Ahin Health Center, Al Multaqa Health Center, Al Tareef Health Center, as well as the Wadi Al Hawasnah and Wadi As Sarmi Hospitals.

### Eligibility criteria

Eligibility criteria required that participants be adults (≥18 years) diagnosed with type 2 diabetes mellitus (T2DM) through clinical evaluation and/or documented evidence and who had lived with the condition for at least one year. Participants also had to provide written informed consent and demonstrate proficiency in reading Arabic. Conversely, the study excluded individuals who declined to participate, those experiencing severe symptoms of illness, and patients with self-reported psychological disorders as defined by the DSM-5 (23).

### Inclusion criteria

- Adults aged 18 years or older.
- Clinically diagnosed with type 2 diabetes mellitus (T2DM), with documented evidence.
- Living with T2DM for at least one year.
- Able to read and understand Arabic.
- Provided written informed consent to participate.

### Exclusion criteria

- Individuals who declined participation.
- Patients with severe physical illness or symptoms at the time of the study.
- Individuals with self-reported psychological disorders, as defined by the DSM-5 (23).

### Sample size calculation

The Cochran formula (24) was used to calculate the sample size of this study, as shown below:


$$n=\frac{Z^{2}P\left(1-P\right)}{\left(\text{e}\right)2}$$

Where;

n= the desired sample size.

Z= level of confidence= 95% CI (1.96).

P= prevalence = 15.7% (25).

e = merging of error =5%; expressed in precision (d=0.05).


$$\frac{{\left(1.96\right)}^{2}\times 0.157\times 0.843}{{\left(0.05\right)}^{2}}=203$$

### Sample and sampling

The sampling approach appears to be a stratified cluster sampling technique. Initially, the governorate was divided into three strata urban, suburban, and rural to capture a diverse array of regional characteristics. Within each stratum, the 30 health institutions served as clusters from which participants were subsequently sampled. The calculated sample size was 203. We increased it to 225 to enhance statistical power and compensate for potential non-responses. Augmenting the sample size enhances the study’s statistical power, hence increasing the probability of detecting genuine relationships within the data. Furthermore, an increased quantity of samples diminishes the possible impact of undesirable readings. It improves the accuracy of the results by rendering them more representative of the total population, therefore augmenting their reliability (26–28).

### Data collection instruments

A self-administered questionnaire was used, consisting of two sections: one for socio-demographic and clinical characteristics, and the other utilizing the Self-Efficacy for Managing Chronic Disease 6 Scale (SEM6S) to assess patients’ confidence in managing their condition. Due to the diversity in educational backgrounds among adult patients with T2DM in North Al Batinah, a self-administered approach was used to enhance respondent anonymity and reduce interviewer bias. The questionnaire was crafted in plain, straightforward Arabic and underwent pilot testing to guarantee comprehensibility across various literacy levels. Trained research assistants were present on site to offer concise instructions and address procedural inquiries, without swaying responses, thereby maintaining the self-report integrity of the instrument while assisting participants needing more clarification. This methodology harmonized rigor with accessibility, enabling all eligible patients to engage easily and confidentially.

*The first section collected socio-demographic and clinical characteristics including* age, gender, marital status, educational attainment, smoking status, family history, chronic diseases, the duration of diabetes mellitus diagnosis, insulin use, and use of oral hypoglycemic agents. Glycated hemoglobin (HbA1c) was categorized as a dichotomous variable: ‘controlled’ if the HbA1c value was less than or equal to 7%, and ‘uncontrolled’ if the value exceeded 7% (29). Certified nurses collected blood samples at the clinic for point-of-care testing. HbA1c was recorded as the patient’s most recent result, which had to have been obtained within the last three months, as documented in the medical record.

*The second section comprised the Self-Efficacy for Managing Chronic Disease 6 Scale (SEM6S)* a tool used to assess individuals’ confidence in performing tasks related to managing chronic diseases. The reliability of the original English version of the Self-Efficacy for Managing Chronic Disease scale reported an internal consistency coefficient of 0.90. On a 10-point scale, the measure includes 6 items ranging from “not at all confident (1)” to “completely confident (10)”. A higher mean score across at least four of the six items indicates greater self-efficacy. The Self-Efficacy for Managing Chronic Disease 6-item Scale generates a total score ranging from 6 to 60. A score of 30 is commonly used as a cut-off point to distinguish between low and adequate self-efficacy levels, as supported by the original scale developers and validated in prior studies (30, 31). The Arabic version mirrors the structure of the original validated scale and demonstrated good reliability (Cronbach’s alpha = 0.80). The Arabic questionnaire was adopted from Allam et al. (32) who previously undertook a meticulous translation and cultural adaption process. Their research encompassed forward and backward translation, expert evaluation, and pilot testing to ascertain the tool’s linguistic validity and cultural appropriateness for Arabic-speaking individuals with chronic illnesses. By employing this previously validated version, the current study guaranteed measurement consistency and comparability with extant research.

### Data collection procedures

The eligible participants attending selected healthcare institutions within North Al Batinah were approached by trained research assistants, informed about the study, and invited to participate upon providing written informed consent. The research focused on Omani patients diagnosed with T2DM who were undergoing medical treatment at healthcare facilities within the North Al Batinah region, encompassing primary and extended health centers as well as public hospitals. In preparation for this phase, the primary investigator organized and oversaw a comprehensive training program for thirteen research assistants, each affiliated with a different healthcare facility. The research assistants were employed to ensure that all participants could fully engage with the questionnaire. The research assistants supported participants by reading the questions aloud. They also helped clarify any unclear items and ensured accurate completion of the questionnaire. The training of research assistants was conducted virtually via a Zoom session, where standardized procedures for administering the questionnaire, obtaining consent, and addressing participant queries were demonstrated and discussed. Following this training, each assistant was assigned to a specific healthcare facility. Their responsibilities and daily targets were clearly outlined in coordination with the North Al Batinah Health Directorate. This ensured that the virtual preparation translated effectively into consistent and standardized in-person data collection.

The research assistants received explicit directives about the target figures for which they were accountable within the overall sample. The questionnaire began with the study’s title and was followed by a comprehensive description of the study’s purpose, potential benefits, risks, and privacy assurances. Participants were informed of their right to withdraw at any time and were told the questionnaire would take approximately 15 to 20 minutes to complete. The study team swiftly addressed any participant queries during the data collection phase. A thorough verification process confirmed the completeness of the data, reinforcing the reliability of the collection methods and the study’s data integrity.

### Data analysis

Data analysis was performed using SPSS version 29.0. Continuous variables were expressed as mean and standard deviation, whereas categorical variables were represented as frequency and percentage. The independent samples t-test was utilized to compare mean scores between two groups, while the one-way ANOVA was employed for mean scores among three or more groups. A p-value below 0.05 was deemed statistically significant.

### Ethical consideration

The research received approval from the International Review Board (IRB) of Central South University [No. E2023193], and from the Medical Research Ethics Committees (MREC) of the Ministry of Health in Oman. To ensure cultural and linguistic appropriateness, Arabic written informed consent was obtained from all participants after being explained verbally by research assistants, who verified they understood the purpose of the study and voluntarily agreed to participate. Confidentiality was strictly maintained, and participant anonymity was ensured during data collection. Consequently, to ensure the security of the data, all hardcopy forms were secured in locked cabinets at the principal investigator’s institution, with access permitted only to the principal investigator and designated research team members. Participation was entirely voluntary, and no incentives were provided. Participants retained the right to withdraw from the study at any time without penalty.

## Results

### General characteristics of the participants

This present study enrolled 225 individuals diagnosed with type 2 diabetes mellitus. Approximately half of the participants were between 51 and 70 years of age, and a substantial majority were married. Fifty percent of the patients were females, and additionally, two-thirds were non-smokers. In terms of education attainment, around half of the participants were illiterate, whereas third had completed only primary education. Additionally, two-thirds of the participants were unemployed, and most of the participants reported a monthly income of less than 500 Omani Rial (**Table 1**).

**Table 1:** Characteristics of patients with T2DM (N= 225).

Socio-demographic variable	n (%)
Age	
18-30 years	13 (5.8)
31-50 years	64 (28.4)
51-70 years	118 (52.4)
>70 years	30 (13.3)
Gender	
Male	100 (44.4)
Female	125 (55.6)
Marital status	
Married	158 (70.2)
Unmarried	67 (29.8)
Smoking status	
Smoker	23 (10.2)
Non-smoker	202 (89.8)
Education level	
Illiterate	104 (46.2)
Primary school	74 (32.9)
Secondary school	35 (15.6)
University education	12 (5.3)
Employment status	
Employed	31 (13.8)
Unemployed	156 (69.3)
Retired	38 (16.9)
Monthly income	
<500 Omani Rial	197 (88.3)
500-999 Omani Rial	23 (10.3)
1000-1500 Omani Rial	3 (1.4)

### Clinical characteristics of the participants

Most participants had a family member affected by diabetes. Approximately half of the participants had been diagnosed with diabetes for over a decade. Among the diabetes medications administered, 138 (61.3%) were oral agents, while 39 (17.3%) were a combination regimen of oral medications. Furthermore, 81.8% of the participants had at least one additional chronic condition. A total of 119 individuals (54.1%) were classified as obese (BMI ≥ 30), whereas 71 participants (32.3%) were classed as overweight (BMI between 25.0 and 29.9) (**Table 2**).

**Table 2:** Clinical Characteristics of patients with T2DM.

Variable	n (%)
A family member suffers from diabetes	
Yes	177 (79.0)
No	47 (21.0)
Number of years with diabetes	
<5 years	45 (20.0)
5-10 years	51 (22.7)
>10 years	129 (57.3)
Medications used for diabetes	
Lifestyle modification	19 (8.4)
Oral medications	138 (61.3)
Insulin	29 (12.9)
Both oral medications and insulin	39 (17.3)
Other chronic disease	
Yes	184 (81.8)
No	41 (18.2)
Patient's place of residence	
Rural or mountainous area	75 (33.3)
The area between rural and urban	76 (33.8)
Urban area	74 (32.9)
Body Mass Index (BMI)	
Underweight (less than 18.5)	2 (0.9)
Average weight (18.5 to 24.9)	28 (12.7)
Overweight (25.0 to 29.9)	71 (32.3)
Obesity (30 or above)	119 (54.1)

### The status of self-efficacy and glycemic control among T2DM

Glycemic control was assessed by hemoglobin A1c. Among the study participants, 70 (31.1%) exhibited good glycemic control, 90 (40.0%) demonstrated moderate control, and 65(28.9%) had poor control. The mean hemoglobin A1c level was 8.23 ± 2.08, indicating moderate glycemic control. The mean self-efficacy score was 29.99 ± 11.41, falling just below the cutoff of 30, suggesting a low self-efficacy level (**Table 3**).

**Table 3:** The status of glycemic control and self-efficacy of T2DM.

Hemoglobin A1c level	Number	Percent
Good (<7%)	70	31.1
Moderate (7-9%)	90	40.0
Poor (>9%)	65	28.9
Total	225	100
Scale		Mean±*SD*
Hemoglobin A1c		8.23±2.08
Self-Efficacy Scale		29.99±11.41

### Association between diabetes self-efficacy and patients’ characteristics

Our findings revealed statistically significant differences in glycemic control across different age groups. Specifically, patients aged 31–50 years had significantly higher mean HbA1c levels compared to older adults, indicating poorer glycemic control (F: 2.313, 95% CI: 0.047–0.304, *p*: 0.015). Furthermore, married participants had significantly higher HbA1c levels than unmarried participants (F = 3.241, 95% *CI*: 0.080–0.335, *p*: 0.025), possibly reflecting the influence of family-related stressors or competing caregiving responsibilities. Additionally, a significant association was found between the type of diabetes medication and glycemic control. Moreover, patients receiving both oral medications and insulin exhibited significantly poorer glycemic control (F: 3.903, 95% *CI*: 0.169–0.419, *p*< 0.001). Finally, the results indicated a significant association between the presence of other chronic diseases and glycemic control, as patients without chronic diseases exhibited poorer glycemic control (F: 3.070, 95% CI: 0.036–0.294, *p*: 0.049). Unexpectedly, patients with no additional chronic diseases had poorer glycemic control. One possible explanation is that patients with comorbidities may engage more frequently with healthcare systems, thus improving treatment adherence (**Table 4**).

**Table 4:** Association between the patient's characteristics and glycemic control.

Variable	Glycemic control (HbA1c)			Effect Size	F	95% CI	Chi-square (χ²)	p-value
	Good (<7%)	Medium (7-9%)	Poor (>9%)					
	n (%)	n (%)	n (%)					
**Gender**				0.084	0..798	0.001 - 0.214	1.596	0.450
Male	35 (35.0)	36 (36.0)	29 (29.0)					
Female	35 (28.0)	54 (43.2)	36 (28.8)					
**Age**				0.175	2.313	0.047 - 0.304	13.876	0.015*
18-30 years	2 (15.4)	6 (46.2)	5 (38.5)					
31-50 years	16 (25.0)	22 (34.4)	26 (40.6)					
51-70 years	38 (32.2)	48 (40.7)	32 (27.1)					
>70 years	14 (46.7)	14 (46.7)	2 (6.7)					
**Marital status**				0.208	3.241	0.080 - 0.335	19.446	0.025*
Married	54 (34.2)	54 (34.2)	50 (31.6)					
Unmarried	16 (23.9)	36 (53.7)	15 (22.4)					
**Smoking status**				0.104	0.814	0.001 - 0.234	4.884	0.476
Smoker	7 (30.4)	7 (30.4)	9 (39.1)					
Non-smoker	63 (31.2)	83 (41.1)	56 (27.7)					
**Education level**				0.153	1.309	0.023 - 0.281	10.473	0.282
Illiterate	37 (35.6)	43 (41.3)	24 (23.1)					
Primary school	23 (31.1)	29 (39.2)	22 (29.7)					
Secondary school	6 (17.1)	15 (42.9)	14 (40.0)					
University education	4 (33.3)	3 (25.0)	5 (41.7)					
**Employment status**				0.071	0.566	0.003 - 0.201	2.265	0.704
Employed	8 (25.8)	11 (35.5)	12 (38.7)					
Unemployed	48 (30.8)	65 (41.7)	43 (27.6)					
Retired	14 (36.8)	14 (36.8)	10 (26.3)					
**Duration of diabetes**				0.089	0.898	0.002 - 0.219	3.592	0.450
<5 years	17 (37.8)	19 (42.2)	9 (20.0)					
5-10 years	16 (31.4)	17 (33.3)	18 (35.3)					
>10 years	37 (28.7)	54 (41.9)	38 (29.5)					
**Diabetic Medication**				0.294	3.903	0.169 - 0.419	39.028	<0.001*
Lifestyle modification	7 (36.8)	5 (26.3)	7 (36.8)					
Oral medications	52 (37.7)	58 (42.0)	28 (20.3)					
Insulin	5 (17.2)	17 (58.6)	7 (24.1)					
Both oral medications and insulin	6 (15.4)	10 (25.6)	23 (59.0)					
**Other chronic diseases**				0.165	3.070	0.036 - 0.294	6.141	0.049*
Yes	58 (31.5)	79 (42.9)	47 (25.5)					
No	12 (29.3)	11 (26.8)	18 (43.9)					
**Patient's residence**				0.714	0.999	0.574 - 0.854	9.959	0.245
Rural or mountainous	23 (30.7)	34 (45.3)	18 (24.0)					
The area between rural and urban	28 (36.8)	23 (30.3)	25 (32.9)					
Urban area	19 (25.7)	33 (44.6)	22 (29.7)					
**Body Mass Index (BMI)**				0.116	0.987	0.001 - 0.247	5.921	0.400
Underweight (less than 18.5)	0 (0.0)	1 (50.0)	1 (50.0)					
Average weight (18.5 to 24.9)	13 (46.4)	9 (32.1)	6 (21.4)					
Overweight (25.0 to 29.9)	19 (26.8)	27 (38.0)	25 (35.2)					
Obesity (30 or above)	35 (29.4)	51 (42.9)	33 (27.7)					

CI, Confidence Interval; F-value: Approximated from the chi-square value using the formula F = χ² / df.

Bold values indicate statistically significant results (p < 0.05).

## Discussion

This study evaluated the socio-demographic and clinical characteristics of 225 Omani patients diagnosed with type 2 diabetes mellitus, highlighting key factors potentially associated with glycemic control. The majority of participants were between 51 and 70 years of age, predominantly married, and nearly half were female. A significant portion of the sample had limited educational attainment; 46.2% were illiterate, and 32.9% had only primary education, an indicator that may reflect limited health literacy that may hinder effective diabetes management. Additionally, low reported income levels could restrict access to healthcare services, medications, and diabetes supplies, contributing to suboptimal glycemic outcomes. These socio-economic patterns are consistent with previous research suggesting that lower education and income levels are associated with poorer diabetes self-management and outcomes (33, 34). This study was conducted in the governorate of North Al Batinah, which, while diverse, may not be representative of the overall Omani population, particularly those residing in highly urbanized areas such as Muscat. Differences in healthcare infrastructure, access, levels of education, and lifestyle patterns across regions may influence diabetes self-management and glycemic control. For instance, urban populations may have more access to specialist services or diabetes education programs, which may result in unequal outcomes.

Clinically, about half of the participants had been living with diabetes for over a decade, and a majority reported a family history of the disease, which may suggest a genetic predisposition. Most were of patients treated with oral hypoglycemic agents (61.3%), while 17.3% received combination therapy with insulin, a pattern that may reflect more advanced disease or increased insulin resistance. The high prevalence of comorbidities (81.8%), overweight (32.3%), and obesity (54.1%) among participants aligns with global findings that associate these factors with more complex diabetes management (35, 36).

Glycemic control, as measured by HbA1c levels, was suboptimal in most participants, with only 31.1% achieving good control and a mean HbA1c of 8.23 ± 2.08. To assess diabetes self-management confidence, the study utilized a validated self-efficacy scale, with scores ranging from 0 to 60. The mean self-efficacy score was 29.99 ± 11.41, falling below the established threshold for adequate self-management confidence. Correlational analysis revealed a negative association between self-efficacy and HbA1c levels, indicating that participants with higher self-efficacy tended to have better glycemic control.Suboptimal glycemic control is often linked to poor adherence to treatment, including irregular use of medications, inconsistent self-monitoring of blood glucose, and non-compliance with dietary recommendations. Poor health literacy or education about the necessity of glycemic control can also be a factor, as Omani patients may not be aware of the long-term implications of poorly controlled diabetes. Socio-economic issues like insufficient access to healthcare services, drugs, or diabetes care programs can also be harmful to disease control. The low self-efficacy scores observed among Omani participants suggest limited confidence in managing their diabetes effectively. This may be due to a lack of structured diabetes education, weak social or clinical support systems, or prior experiences with uncontrolled blood glucose levels. This finding aligns with existing literature suggesting that self-efficacy is a key psychological determinant of diabetes self-care behaviors, including medication adherence, dietary regulation, and blood glucose monitoring. Low self-efficacy in this cohort may reflect limited access to structured diabetes education, insufficient support systems, or prior challenges in managing blood glucose, all of which can undermine confidence and contribute to poorer outcomes (37, 38).

Subgroup analysis revealed that patients aged 31–50 had significantly poorer glycemic control (*p* = 0.015), which may reflect challenges unique to this age group, such as work-related stress and competing responsibilities. Married individuals also showed lower control (*p* = 0.025), possibly due to lifestyle or family-related factors. Patients on combination therapy had worse glycemic outcomes, which may be indicative of more advanced disease or greater insulin resistance The finding that patients receiving both oral medications and insulin had significantly poorer glycemic control may indicate a more advanced disease stage or higher insulin resistance, necessitating more aggressive management strategies. The increasingly impaired glycemic control in Omani patients on oral agents, as well as insulin, might be an indicator of a more advanced stage of diabetes or greater insulin resistance. The ability of the body to produce sufficient insulin or respond to insulin diminishes to the point that both oral agents and insulin become necessary to keep blood glucose within normal limits. This combination therapy is usually employed when oral therapy by itself is no longer adequate to maintain glycemic control in a satisfactory range (39). Insulin resistance, a feature of advanced type 2 diabetes, can also be a cause of this syndrome because it renders the body less sensitive to insulin, requiring greater dosages or polypharmacy regimens to be controlled (40). Delayed diagnosis, unhealthful lifestyle intervention, or failure to adhere to treatment regimens are other disease-causative factors in Omani patients.

Participants without additional chronic diseases exhibited poorer glycemic control (*p* = 0.049). While this finding may appear unexpected, it is important to interpret it cautiously. While seemingly counterintuitive, this result can be explained by several interconnected factors. Firstly, individuals with multiple chronic diseases are more likely to utilize healthcare services more frequently, with close clinical surveillance and regular follow-up (41). This greater exposure can make timely changes in diabetes treatment strategies easier to implement (42). Second, when there is more than one condition, there is usually more need for polypharmacy, which can inspire patients to utilize more rigorous medication adherence practices and an increased sense of their health status (43). These patients will also possibly be given more extensive care plans, focusing on lifestyle changes, dietary limitations, and self-monitoring activities, thereby supporting improved glycemic control. Although this finding contradicts some of the previous evidence that comorbidities are challenging for glycemic control, it points toward the necessity for patient-centered, integrated care models (44). Future work should be done on this relationship with longitudinal data to establish causality and to know if taking advantage of care for comorbid conditions could improve diabetes outcomes in more general patient populations.

## Strengths and limitations

The study demonstrates several notable strengths. The recruitment of a representative sample from multiple healthcare facilities within the North Al Batinah region ensures that the findings are contextually relevant to the Omani population with T2DM. The utilization of validated instruments such as the Self-Efficacy for Managing Chronic Disease Scale (SEM6S) and objective measures like HbA1c further strengthened the internal validity and reliability of the data. Additionally, the rigorous data collection process, which included a meticulous verification procedure to confirm the absence of missing data, emphasizes the methodological robustness of the study. Despite the latter strengths, the study is subject to several limitations. The cross-sectional design of this study limits the capacity to establish causal relationships or monitor changes in self-efficacy and glycemic control over time; therefore, we suggest longitudinal follow-ups. The findings may possess restricted generalizability, as data were exclusively gathered from North Al Batinah, perhaps failing to reflect the wider Omani community. Ultimately, significant behavioral aspects including diet, physical exercise, and psychological impacts were not assessed, potentially impacting the interpretation of glycemic control results. Future investigations should rectify these deficiencies by employing longitudinal methodologies and expansive, multi-regional sampling. Finally, future research should strive for multi-center studies with varied geographic and socio-economic sites across Oman to enhance the representativeness and external validity of the findings.

## Conclusion

Glycemic control in patients who had T2DM was markedly affected by socio-demographic factors, especially age and marital status. Notably, people with concomitant chronic illnesses demonstrated superior glucose management. This research suggests that the existence of supplementary health conditions enhances health awareness and compliance with medical protocols, either due to heightened engagement with healthcare professionals or more extensive treatment strategies. Instead of obstructing glycemic control, comorbidities may act as a stimulus for more organized and attentive illness care. These results highlight the intricate, multifaceted nature of glycemic management, influenced by clinical and psychological aspects as well as individual social situations. Customized therapies that consider these factors are crucial for enhancing diabetes management. Future research should employ longitudinal study designs to elucidate the direction and causality of the observed connections, especially between self-efficacy and glycemic outcomes. Examining the impact of comorbidities on patient behavior and care involvement may guide the creation of focused interventions to enhance outcomes among varied patient populations.

## Data Availability

The raw data supporting the conclusions of this article will be made available by the authors, without undue reservation.
